# Prosthetic Management of a Child with Hypohidrotic Ectodermal Dysplasia: 6-Year Follow-Up

**DOI:** 10.1155/2016/2164340

**Published:** 2016-10-16

**Authors:** Antonione Santos Bezerra Pinto, Moara e Silva Conceição Pinto, Cinthya Melo do Val, Leonam Costa Oliveira, Cristhyane Costa de Aquino, Daniel Fernando Pereira Vasconcelos

**Affiliations:** ^1^Department of Morphology, Faculty of Medicine, Federal University of Ceará, Fortaleza, CE, Brazil; ^2^Department of Histology and Embryology, Faculty of Biomedicine, Federal University of Piauí, Parnaíba, PI, Brazil; ^3^Department of Genetic and Applied Toxicology, Lutheran University of Brazil, Canoas, RS, Brazil; ^4^Department of Medical Skills, Faculty of Medicine, Federal University of Piauí, Parnaíba, PI, Brazil; ^5^Laboratory of the Biology of Tissue Healing, Ontogeny and Nutrition, Department of Morphology and Institute of Biomedicine, School of Medicine, Federal University of Ceará, Fortaleza, CE, Brazil

## Abstract

Ectodermal dysplasia (ED) is a genetically heterogeneous condition resulting from clinical anomalies of structures derived from the ectoderm, such as the hair, nails, sweat glands, and teeth. This clinical report presents the case of a child diagnosed with hypohidrotic ED at 2 years of age; clinical and imaging evaluation was performed with 6-year follow-up, and we present details of the prosthetic dental care, with a 12-month follow-up. The patient's masticatory capacity had improved, leading to the child gaining 4 kg. In conclusion, prosthetic management was noninvasive and appeared to lead to developmental benefits for the patient.

## 1. Introduction

Ectodermal dysplasia (ED) comprises a large group of clinical and genetically heterogeneous diseases affecting the ectoderm-derived structures, such as the hair, nails, sweat glands, and teeth [1]. The prevalence is estimated between 1,6 and 22 per 100,000 persons [2].

The most common forms of ED are the X-linked hypohidrotic (Christ-Siemens-Touraine syndrome) and hidrotic (Clouston syndrome) types. The former presents with hypodontia or anodontia, hypotrichosis, and hypohidrosis or anhidrosis, while the latter is more severe, involving nail dystrophy, hypotrichosis, and palmoplantar keratoderma [3, 4].

Prosthetic dental treatment for ED is necessary to improve chewing, facial aesthetics, speech, nutrition, and social integration, the last of which can influence the patient's emotional health [5]. For these reasons, the technique is widely discussed in the literature. The therapy must be adapted to each case and varies from simple restorations to prostheses and implants [6–9].

Herein, we report the case of a child with hypohidrotic ED (HED) who underwent imaging and prosthodontic treatment and was followed up for 6 years; we also examine the influence of the procedure on the child's development, consider his ability to contribute to clinical management at an early age, and discuss tailoring oral rehabilitation to the idiosyncrasies of each patient's facial bone with similar facial anomalies.

## 2. Clinical Report

The patient began visiting the dentist at 2 years of age. At this time, his mother complained that several teeth had not erupted.

The mother described several episodes of hyperthermia, lack of sweating, crying with few tears, constant water intake, and frequent bathing. During viral infections, the mother had observed gelatinous, yellow-brown secretions from the respiratory tract. No anomalies were reported concerning the child's neurological development. He had been attending pediatric appointments, but the condition had never been suspected.

**Figure 1:**
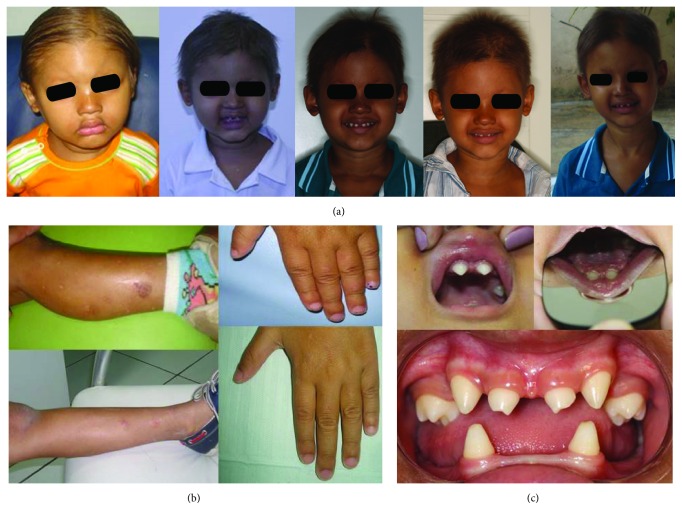
(a) Patient's visage at 2, 3, 4, 5, and 6 years of age with sparse hair, frontal bossing, saddle nose, and everted prominent lips. (b) Bright, dry skin on the leg; normal nails. (c) Intraoral examination showing variation in the timing and shape of the teeth, hypodontia, and ogival palate.

After anamnesis, wherein the boy's medical history was recalled by his mother, both extra- and intraoral examinations were performed (Figure 1); on this basis, the child was diagnosed with HED.

Initial dental treatment was based on maintaining oral health, which involved instructing both the boy and his mother in oral hygiene in order to preserve the teeth against oral diseases such as caries and periodontal disease. The mother was further instructed to seek the advice of a medical geneticist who would confirm the diagnosis of HED, request additional tests, and contribute to the patient's treatment.

**Figure 2:**
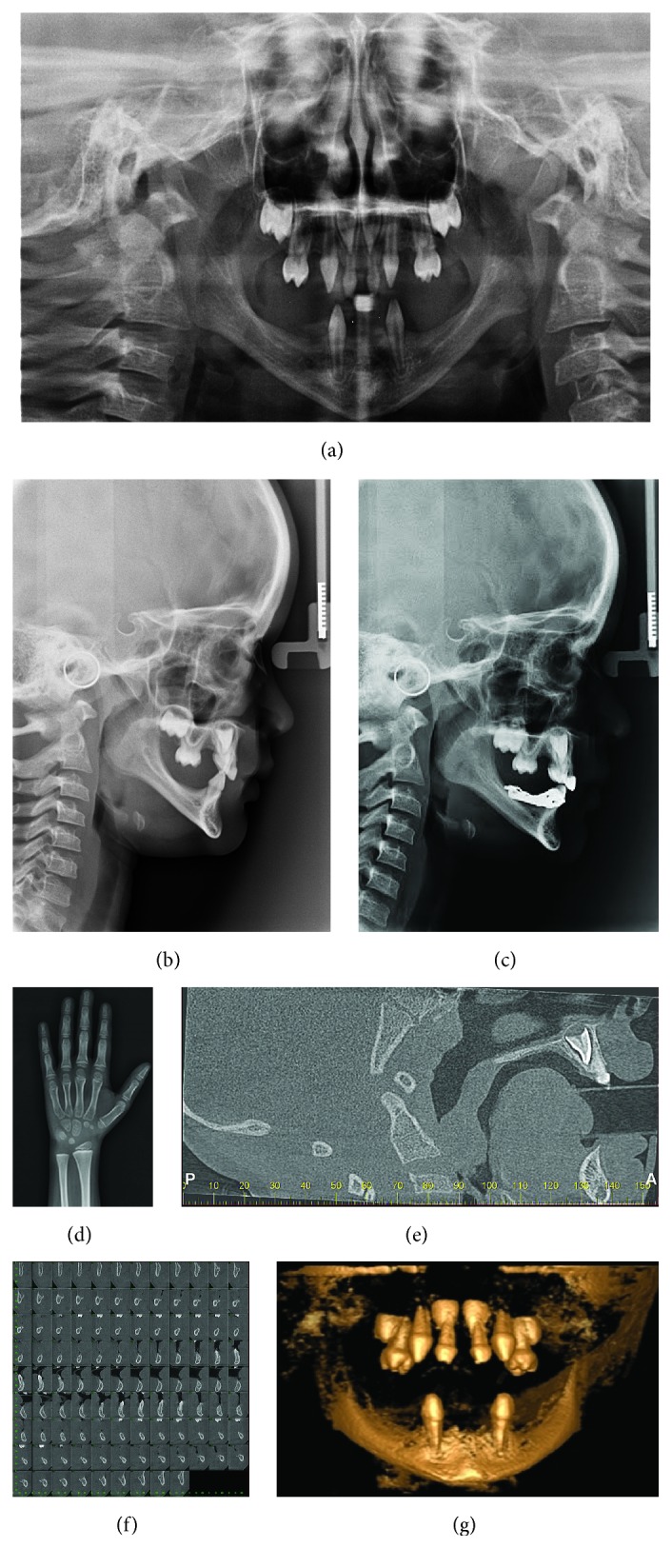
(a) Panoramic radiograph. (b) Lateral cephalogram showing decreased mandibular length and mandibular anterior facial height. (c) Lateral cephalogram taken 6 months after the patient began prosthetic rehabilitation. The image shows the growth pattern between the bone bases. (d) Carpal radiograph at 6 years of age matching the chronological age. (e) Computed tomography (CT) sagittal cut evidencing the presence of hypertrophic tonsils, consistent with recurrent infections of the lower respiratory tract. (f) Cross-sectional CT images showing reduced height and thickness of the alveolar bone, as well as mandibular micrognathia. (g) 3D reconstruction.

From the age of 3 to 6 years, the child periodically attended clinical and imaging exams (Figures 1 and 2) to assess bone development and identify when to begin oral rehabilitation.

When the patient was 6 years old, a cephalometric analysis was carried out (Figure 2(b)) revealing that he had a convex profile, as well as a Class I skeletal malocclusion. Using the Eklof and Ringertz index and a carpal radiograph (Figure 2(d)), we determined that the patient's bone age matched his chronological age. What is more, cone-beam computed tomography (CBCT) revealed both hypertrophy in the tonsils and mandibular micrognathia (Figures 2(e) and 2(f)).

The patient was of school age and was willing to cooperate with treatment, especially since the characteristic phenotype of the syndrome, and in particular the dental condition, was affecting him emotionally—he felt different from his classmates.

Informed consent was given by the boy's mother, and the treatment consisted of restorations of the upper and lower teeth and the installation of a lower removable partial denture (Figure 4) when the boy was 7 years old.

**Figure 3:**
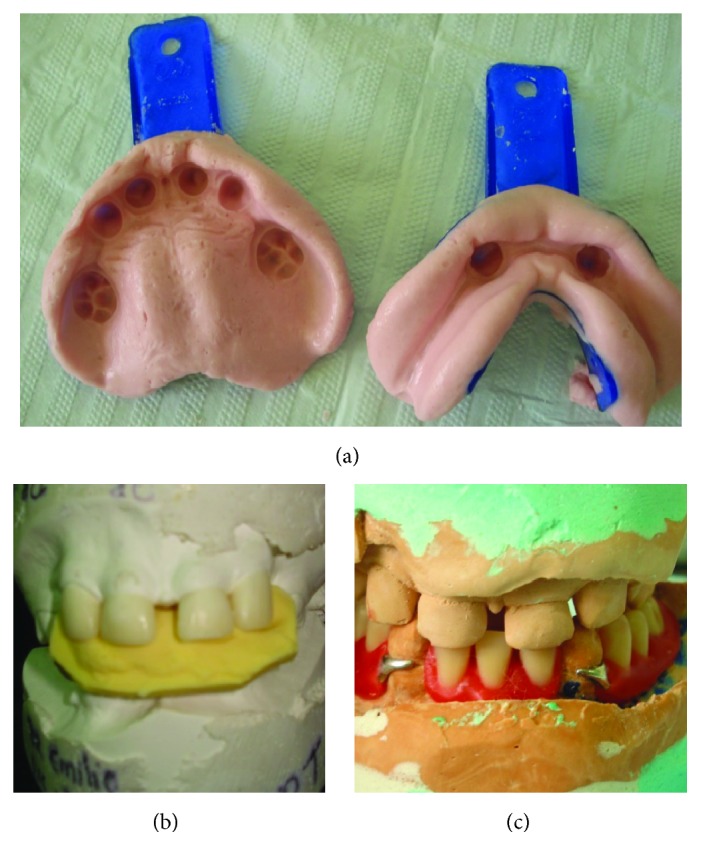
Stages of prosthetic rehabilitation. (a) Upper and lower impression. (b) Template for maxillary tooth restorations. (c) Mandibular partial dentures.

An impression was taken using alginate (Figure 3(a)) (Jeltrate® Plus™; Dentsply) to obtain study cast models (Durone IV Salmon®; Dentsply). From these, diagnostic wax-up of the upper anterior teeth was created to facilitate planning of the lower prosthesis.

A further impression of the diagnostic wax-up was made, using addition silicon (Adsil®; Vigodent) to construct a template that would guide restorations of the deciduous teeth (Figure 3(b)). The maxillary conoid teeth were reconstructed by increasing their dimensions, but maintaining the spaces characteristic of the deciduous dentition. The restorations were made using composite resin AO.5 (Opallis®; FGM) so as to be white and opaque.

The lower partial denture (Figure 3(c)) was supported by the mandibular primary canines, which received increments of resin A3 (IPS Empress® Direct™; Ivoclar Vivadent), so as to resemble the premolars. The appropriate tooth color was then selected (tooth color 60; SPG pop) and the gum was characterized (number 15; Tomaz Gomes System, VIPI).

At the installation of the prosthesis, both the patient and his mother were instructed regarding prosthesis placement, removal, and hygiene.

Adjustments were made 48 hours after installation. The prosthesis showed good retention, and the patient adapted well to it. Then, the next visit was scheduled at 6 months for follow-up and the patient's mother was advised to arrange additional visits in the case of complications.

**Figure 4:**
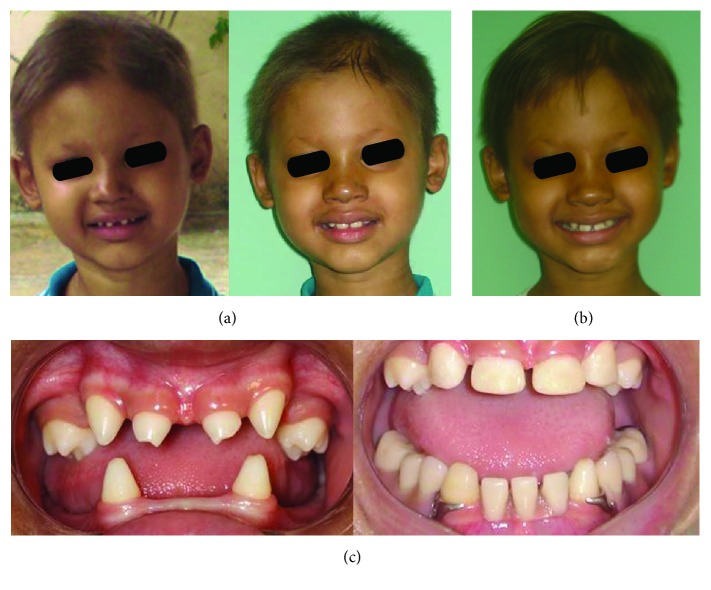
Patient before and after treatment. (a) Initial and final facial aspect. (b) 12 months after the installation of the prosthesis. (c) Oral aspects.

Six months after installation of the prosthesis, the periodontal condition of the teeth, occlusion, functional adaptation, and cephalometrics were evaluated (Figure 2(c)). Clinical evaluation was repeated over six months when the patient was 8 years old (Figure 4(b)) and new adjustments in prosthesis were performed.

According to his mother, the child's chewing ability had improved and he was able to enjoy foods that he could not chew before, such as meat, cheese, and fibrous vegetables. This resulted in a gain of 4 kg—from 16 kg to 20 kg—during the first 6 months of adaptation.

Both the family and the teacher noticed the behavioral differences, and the classmates approved the child's new appearance, which suggested impact on his socialization.

It must be noted that the patient's mother, as his legal guardian, read and signed an informed consent form authorizing the use of data, images, and all information relating to the dental patient follow-up for use in publications and/or scientific events.

## 3. Discussion

Important dental defects are associated with the syndrome. During childhood particularly, this condition is a major cause of frustration that impacts the intellectual and psychological maturity of the patient [5, 6].

Many children require extensive treatment; however, treatment cost should be evaluated. All changes in the dental arches, including alveolar bone growth in response to tooth eruption, should be monitored to ensure appropriate adjustments are made. Long-term treatment is an active process that must be constantly adapted to the child's development and growth [10].

In the present case, after the prosthetic rehabilitation, the child experienced a wider variety of foods. The relationship between the upper and lower jaws, esthetics, and self-esteem were all improved. Children with these prostheses should perform standard oral hygiene and take care of the devices by themselves.

ED treatment using removable partial dentures is indicated for children and adults [6, 7]. Nonetheless, the use of partial dentures has been described in a 2-year-old HED patient; such early rehabilitation prevents growth abnormalities and improves socialization [7].

The installation of implants in adult ED patients has been widely reported as a treatment option with high success rate and the associated benefits of rehabilitation [8]. Nonetheless, the high cost, possible failure of osseointegration, and need for complete bone growth are disadvantages, particularly if dental implants are placed before dental and skeletal maturation [9].

In this case, dental implants were not indicated, because CBCT (Figures 2(e) and 2(f)) revealed both maxillary and mandibular micrognathism caused by the absence of teeth. The cephalometric analysis (Figures 2(b) and 2(c)) indicated that the patient should be followed up to assess bone growth after placement of the prosthesis.

After prosthetic rehabilitation, masticatory function improves, as in the present case, where the patient was able to eat meat, fish, fibrous vegetables, and cheese. In general, dentures are well accepted by parents and patients, and the psychological benefits after oral rehabilitation are many [5–7]. The authors are unanimous in recommending early prosthetic treatment in patients with this syndrome, especially when the child already has a social life, as is the case with children of school age.

## 4. Conclusion

A removable partial denture and restoration were a noninvasive, reversible, cost-effective, viable, and efficient treatment for a child with hypodontia and HED. Moreover, the treatment appeared to lead to marked developmental benefits for the patient.
